# Bayesian Pathway Analysis of Cancer Microarray Data

**DOI:** 10.1371/journal.pone.0102803

**Published:** 2014-07-18

**Authors:** Melike Korucuoglu, Senol Isci, Arzucan Ozgur, Hasan H. Otu

**Affiliations:** 1 Department of Computer Engineering, Bogazici University Bebek, Istanbul, Turkey; 2 Scientific and Technological Research Council of Turkey (TUBITAK), Informatics and Information Security Research Center, Gebze, Kocaeli, Turkey; 3 Department of Genetics and Bioengineering, Istanbul Bilgi University Santral Campus, Eyup, Istanbul, Turkey; 4 Department of Electrical Engineering, University of Nebraska-Lincoln, Lincoln, Nebraska, United States of America; Memorial Sloan Kettering Cancer Center, United States of America

## Abstract

High Throughput Biological Data (HTBD) requires detailed analysis methods and from a life science perspective, these analysis results make most sense when interpreted within the context of biological pathways. Bayesian Networks (BNs) capture both linear and nonlinear interactions and handle stochastic events in a probabilistic framework accounting for noise making them viable candidates for HTBD analysis. We have recently proposed an approach, called Bayesian Pathway Analysis (BPA), for analyzing HTBD using BNs in which known biological pathways are modeled as BNs and pathways that best explain the given HTBD are found. BPA uses the fold change information to obtain an input matrix to score each pathway modeled as a BN. Scoring is achieved using the Bayesian-Dirichlet Equivalent method and significance is assessed by randomization via bootstrapping of the columns of the input matrix. In this study, we improve on the BPA system by optimizing the steps involved in “Data Preprocessing and Discretization”, “Scoring”, “Significance Assessment”, and “Software and Web Application”. We tested the improved system on synthetic data sets and achieved over 98% accuracy in identifying the active pathways. The overall approach was applied on real cancer microarray data sets in order to investigate the pathways that are commonly active in different cancer types. We compared our findings on the real data sets with a relevant approach called the Signaling Pathway Impact Analysis (SPIA).

## Introduction

Bayesian Network (BN) models have gained popularity for learning biological pathways from microarray gene expression data [1], [2]. BNs represent dependency structure for a set of random variables using directed acyclic graphs and have been used with increasing popularity in mathematics and computational sciences over the past 20 years. However, current BN applications are limited to structure learning using observed data and therefore work only on a few hundreds of variables as structure learning algorithms are computationally complex. This, in turn, results in inefficient use of HTBD, which contain a much larger number of variables.

From a life sciences perspective, data analysis results make most sense when interpreted within the context of biological networks and pathways. Previously established individual gene analysis based methods have been extended to network and pathway scale mostly along the lines of gene set analysis (GSA) [3], [4] or Gene Ontology (GO) based approaches [5]–[7], which focuses on determining predefined gene sets or classes that are significantly regulated. However, these approaches consider the input genes and the target gene sets and classes simply as *lists* and do not incorporate in their models the topology via which genes in these classes interact with each other. Other popular commercial approaches, such as the Ingenuity Pathway Knowledge Base (Ingenuity Inc., California) or PathwayAssist (Ariadne Genomics, California) also identify known pathways as active based on HTBD simply by considering the number of genes shared by the input list and the target pathway. All aferomentioned methods use some variation of the main idea that a functional class is relevant to the observed HTBD if the class possesses a statistically significant amount of the input gene list.

We have recently proposed an approach, called Bayesian Pathway Analysis (BPA), for analyzing HTBD using BNs [8]. In the BPA framework known pathways are modeled as BNs and the processed HTBD is used to score each network to assess its fitness to the observed data; achieving a workflow that incorporates in its model the topology of the pathways. There have since been approaches that model the pathway topology to some degree in the analysis of HTBD [9]–[14]. In terms of general applicability and direct relation to the output of BPA, we have used the Signaling Pathway Impact Analysis (SPIA) [15] in our comparisons. SPIA combines the GSA based pathway activation measure with a novel pathway perturbation score, which reflects the degree to which the deregulation of the genes in the pathway is in concordance with the signaling hierarchy.

In the BPA approach, pathways are retrieved from the KEGG database [16]. Each entry (node) in the pathway is mapped to an internal unique ID and a conversion module carries out the necessary mapping between the input gene expression IDs and the pathway node IDs. Repeating entries in the pathway are merged and represented as a single node while conserving edge relations. BN theory utilizes Directed Acyclic Graphs (DAG) but there may exist cycles in the biological pathways. This is overcome using Spirtes' method where graph representations of structural equation models [17] are converted to collapsed acyclic graphs such that d-separations in the collapsed graph entail the same independency relations defined by the model. To this end, a biological pathway is modeled as a BN, which now can be tested against input data to assess its fitness.

BPA assumes a two-group (e.g. case vs. control) normalized gene expression data as input. The observation matrix to score each DAG is obtained by generating the fold change (FC) values for each pair of samples in the two groups. In this matrix, columns represent genes in the DAG and rows represent pairwise comparisons. If there are *N_1_* and *N_2_* samples in the two groups, the observation matrix consists of *N_1_×N_2_* rows. Each column represents the FC for the corresponding gene in each of the *N_1_×N_2_* pairwise comparisons. These continuous FC values are discretized using a cut-off of 2. If the FC value is greater than 2 or less than 0.5 (i.e. the gene is deregulated), it is converted into 1, and otherwise it is converted into 2.

The degree to which a pathway explains given HTBD is measured using the Bayesian Dirichlet equivalent (BDe) score with equivalent sample size method [18]. In this phase, the BN is updated with the observation matrix during the score calculation. Statistical significance of this measurement is assessed by testing it against datasets generated by applying randomization via bootstrapping where the observed score is ranked against scores obtained from randomized data sets. Bootstrapping is applied to the columns of the observation matrix providing a randomization of the rows, which are used in scoring. The results are evaluated in terms of nominal p-values and false discovery rate (FDR) values correcting for multiple hypotheses testing.

In this paper, we have two fundamental aims. Our first aim is to improve on the BPA system by using the following strategies. In order to optimize the discretization phase, we tried Equal Width, Equal Frequency, K-means, Column K-means, Bi-directional K-means, and Automatic Threshold Discretization [19], [20] in addition to the hard-cut-off levels offered by BPA. In the scoring phase, we applied Akaike Information Criterion (AIC) [21], Bayesian Information Criterion (BIC) [22], and Factorized Normalized Maximum Likelihood (fNML) [23] and compared the results with the BDe scoring scheme. The significance assessment phase was changed so that random data sets were obtained at the gene signal level. In this approach, samples in each of the two classes are randomly permuted to provide new data sets [24]. Each new data set (with new class assignments for each sample) is run through the complete workflow and a score value is calculated. This way, we overcome the cases where the current BPA approach fails to provide randomized data sets. In testing these new approaches, we generated synthetic microarray data that simulates gene expression from *N* pathways where a subset, *N_a_*, of these pathways is active. A performance criterion is assessed by the accuracy of predicting active and passive pathways. In addition to improving the memory and CPU usage of the algorithm, we also added new organisms for which the BPA system can be used and we provide a web portal at http://bioinfo.unl.edu/bpa/ which hosts the stand-alone version of the optimized software along with a tutorial and example data sets.

Our second aim in this study is to apply the improved pathway analysis approach on real cancer data sets. For this purpose, we downloaded real microarray data sets from the NCBI's GEO database regarding bladder, brain, breast, colon, liver, lung, ovarian and thyroid cancers. We investigated the pathways that are commonly identified as active in these various cancer microarray data sets.

## Methods

### Class Label Permutation

In the original BPA system, the observation data matrix for BN scoring is composed of the 2-level discretized FC levels for the genes in the network to be scored. The degree to which a pathway explains given HTBD is measured using the “Bayesian Dirichlet equivalent” (BDe) score and the statistical significance of this measurement is assessed by randomization via bootstrapping where the observed score is ranked against scores obtained from randomized data sets. Randomized data sets are obtained by changing the structure of the columns of the observation matrix via sampling with replacement of each column separately.

In Table 1, we show two sample instances of such input matrices. Here, columns denote the genes and rows denote the pairwise comparison of the samples in the two sample groups (e.g. cancer vs. normal). The aferomentioned randomization method (originally employed by BPA) works successfully when an observation matrix as in Table 1 (a) is the case where a given column does not consist only of one type of observation. However, if the observation matrix turns out to be as in Table 1 (b), where columns represent only one type of observation, randomizing the columns of the observation matrix will not result in any change. Therefore, the scores obtained by randomized data sets will be the same, making the significance assessment almost impossible to achieve. It is possible to obtain matrices as in the latter case, i.e. a matrix where a given column consists only of the same level, when a gene shows the same degree and direction of change between the two classes. In other words, if a gene in a given pathway is consistently 2 or more FC upregulated in one class versus the other, we would end up having the column for this gene to consist only of the same discretization level.

**Table 1:** Sample Observation Matrices.

(a)	G_1_	G_2_	G_3_	G_4_	(b)	G_1_	G_2_	G_3_	G_4_
**O_1_**	1	2	1	1	**O_1_**	1	2	2	1
**O_2_**	1	1	2	1	**O_2_**	1	2	2	1
**O_3_**	2	2	2	2	**O_3_**	1	2	2	1
**O_4_**	1	2	2	2	**O_4_**	1	2	2	1
**O_5_**	2	1	2	1	**O_5_**	1	2	2	1
**O_6_**	2	2	1	1	**O_6_**	1	2	2	1
**O_7_**	1	1	2	1	**O_7_**	1	2	2	1

Columns denote genes/nodes; rows denote observations.

In order to overcome this problem, we applied the permutation method previously described to randomize gene expression data sets [24]. This randomization is done by replacing the samples of each class randomly. Suppose we have a dataset composed of 10 normal and 10 cancer samples. In one instance of the permutation, for example, 3^rd^, 5^th^, and 6^th^ normal samples are replaced with 1^st^, 7^th^, and 9^th^ cancer samples. The observation matrix is generated by pairwise comparison of the signal values over the new order of two classes followed by discretization. This procedure is repeated *B* times and pathway scores are calculated using the discretized matrices. As a result, the statistical significance of the observed score can be assessed accurately via ranking against scores obtained from different observation matrices generated by these *B* randomized data sets. If the score of a given pathway is Sn, its p-value is assessed using 
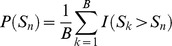
where *I(a)* is 1 if *a* is “true” and 0 otherwise. The significance of each pathway is reported as this nominal p-value and the corresponding false discovery rate (FDR) calculated using the Benjamini-Hochberg procedure [25]

### Discretization

BPA utilized a discretization method such that the continous FC value is represented as 1 if it's greater than 2 or less than ½ (i.e. a gene is dysregulated), and as 2 otherwise. Another use of the 2-level discretization is choosing a cut-off value of 3, i.e., the FC is represented as 1 if its value is greater than 3 or less than 1/3 and as 2 otherwise. In 3-level discretization with the cutoff value 2, the fold change is represented as 1 if its value is greater than 2, as 2 if less than ½, and as 3 otherwise. In 3-level discretization with the cut-off value of 3, the fold change is represented as 1 if its value is greater than 3, as 2 if less than 1/3, and as 3 otherwise.

In this study, we propose new discretization methods [19], [20] to be utilized in processing the observed fold change values for use by Bayesian scoring metrics. An *N*-by-*M* matrix *E* is used to denote the observed FC matrix, where *N* is the number of pairwise comparisons and *M* is the number of genes. *E(n,m)* denotes the FC value of comparison *n* for the gene *m*. *E(n,:)* denotes FC data of comparison *n* for all genes, and *E(:,m)* denotes the FC data of gene *m* for all the comparisons.

#### Equal Width Discretization (EWD)

EWD divides the observation matrix row *n* into *k* intervals of equal width between *E(n,:)_min_* and *E(n,:)_max_*. Thus the intervals of comparison *n* have width *w* =  *(E(n,:)_max_* - *E(n,:)_min_)*/*k*, with boundary points at *E(n,:)_min_ +w, E(n,:)_min_ +2w, …, E(n,:)_min_ + (k - 1)w* where *k* is a positive integer.

#### Equal Frequency Discretization (EFD)

EFD divides the sorted *E(n,:)* into *k* intervals so that each interval contains the same number of FC values.

#### K-means Discretization

K-means divides *E(n,:)* into *k* intervals by k-means clustering so that similar FC values of comparison *n* are placed in the same interval.

#### Column K-means Discretization (Co-k-means)

Co-k-means divides *E(:,m)* into *k* intervals by k-means clustering so that similar FC values for the gene *m* are placed in the same interval.

#### Bidirectional K-means Discretization (Bi-k-means)

In the bi-k-means method both k-means and co-k-means are respectively implemented with parameter *k+1*, giving every FC value two discretized values. If the product of the two values is equal to or greater than *x^2^*, and less than *(x+1)^2^*, the final discretized value of this expression value is *x*, where *x* is a positive integer ranging from *1* to *k*.

#### Automatic Threshold Discretization

There are two options for the automatic threshold discretization, which iteratively determines the cut-off values by minimizing the variance. The whole FC data *E* is divided into two intervals according to a certain cut-off value in the global option. The local option of this method divides *E(:,m)* into two intervals according to the cutoff values defined for each column (gene) separately.

### Scoring

In addition to the BDe scoring scheme, we propose the following score metrics to be used in the BPA system.

#### Akaike Information Criterion (AIC)

AIC is one of the most commonly used information criteria, which selects the model that minimizes the negative likelihood penalized by the number of parameters [21]:
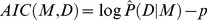
where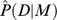 is the maximum likelihood of the model *M*, *D* is observed data, and p is the number of parameters in the model.

#### Bayesian Information Criterion (BIC)

BIC is another widely used information criteria and unlike AIC, BIC is consistent and improves in performance with large sample sizes [22]. BIC is defined as:
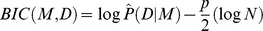



BIC differs from AIC only in the second term, which depends on the sample size *N*.

#### Factorized Normalized Maximum Likelihood (fNML)

Silander et al. [23] developed the fNML score based on the normalized maximum likelihood (NML) distribution [26], [27]. Given a data set *D*, the NML model selection criterion chooses the model *M* for which 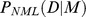 is largest. 
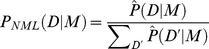
where the normalization is done over all data sets *D′* of the same size as *D*. After taking the logarithm, the score is in a form of penalized log-likelihood given *G* =  {*G_1_*,…,*G_m_*} as the parent set in the DAG (i.e. *G_i_* is the parent set of the node *X_i_* in the DAG):
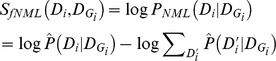
where the normalizing sum goes over all the possible *D_i_* column vectors. Even though the penalty term has an exponential number of terms, it can be evaluated efficiently using a linear-time algorithm introduced in [28]. By calculating the penalty term for each variable in the dataset, the NML becomes factorized.

### Data Sets

We generated synthetic transcriptional regulatory networks and produced simulated gene expression data with noise using SynTReN v1.12 [29]. We created 55 synthetic networks that mimic biological pathways with sizes ranging from 7 to 200. We randomly selected 20 out of 55 pathways to be active and SynTReN generated the corresponding expression datasets for 20 test and 20 control samples with 2249 genes adding a 4% noise level.

To test the optimized and improved BPA performance on real data sets, we used 1 bladder, 2 brain, 2 breast, 1 colon, 2 liver, 1 lung, 1 ovarian, and 2 thyroid cancer data sets. In choosing the data sets, we fixed the platform to be Affymetrix to prevent bias and used data sets where tumor and normal samples are clearly defined and the cancer samples are as homogenous as possible. Most of the chip data came from the Affymetrix HG-U133 Plus 2.0 GeneChip, which is composed of more than 54,000 probe sets representing over 47,000 transcripts providing a comprehensive picture of the human transcriptome. Other chip types include HG-U133A and HG-U133A_2, which represent approximately 22,000 probesets. Prior to application of the proposed approach, raw microarray data has been normalized using Affymetrix Microarray Analysis Suite (MAS) 5.0 algorithm [30].

For each data set, we applied the proposed analysis method with 1000 permutations and assessed significant pathways with a nominal p-value of 0.05 and an FDR of 0.25.

## Results

In Table S1, we list the accuracy levels (if a network is correctly called active/inactive) of the different discretization schemes for 10 simulated datasets (D_1_–D_10_). According to the simulation results, the best discretization method is the 2-level k-means discretization applied to the rows of the observation matrix. This approach achieves an accuracy of 0.962±0,031. Therefore, 2-level k-means method is used as the discretization method for the experiments to determine the best scoring criterion.

The datasets, which are used for the performance measurement of discretization methods, are also used for the assessment of the scoring methods. The obtained prediction accuracies are listed in Table 2. According to the simulation results, the best scoring method is the fNML method, which estimates whether a pathway is active or not with an accuracy of 0.984±0,016. Therefore, the 2-level k-means discretization and fNML scoring methods are used for the real microarray data analysis as this combination achieved the highest accuracy.

**Table 2:** Prediction accuracy of different scoring methods on synthetic datasets.

	BDe	AIC	BIC	fNML
**D_1_**	0.945	0.964	0.909	1.000
**D_2_**	0.982	1.000	0.927	0.964
**D_3_**	0.982	1.000	0.945	1.000
**D_4_**	0.964	0.982	0.982	1.000
**D_5_**	0.945	1.000	0.891	1.000
**D_6_**	0.945	0.982	0.982	0.964
**D_7_**	0.982	0.982	0.927	0.982
**D_8_**	1.000	0.982	0.964	0.982
**D_9_**	0.982	0.982	0.927	0.982
**D_10_**	0.891	0.945	0.945	0.964
**Avg**	0.962	0.982	0.940	**0.984**
**SD**	0.031	0.017	0.030	**0.016**

*BDe: Bayesian Dirichlet Equivalent; AIC: Akaike Information Criterion; BIC: Bayesian Information Criterion; fNML: factorized Normalized Maximum Likelihood*.

In Table 3, we list the 12 real cancer microarray data sets (GEO Numbers, cancer types, and numbers of samples) and the number of pathways identified as active by BPA and SPIA analyses. In Tables S2 and S3, we list the complete list of pathways deemed active by the BPA and SPIA methods for each real cancer microarray dataset, respectively. In total, BPA identified 171 pathways that have been found significant in at least one of the data sets. 15 of these pathways have been found to be significant in at least half of the data sets and therefore potentially represent mechanisms common to different cancer types (see Table S2).

**Table 3:** Cancer Data Sets and numbers of active pathways Identified by BPA and SPIA analyses.

GEO# (GSE)	Cancer Type	Chip Type (HG-U133)	# of Samples	BPA	SPIA
**7476**	bladder	Plus2	12 (9C, 3N)	57	40
**12907**	brain	A	25 (21C, 4N)	81	23
**15824**	brain	Plus2	35 (30C, 5N)	46	32
**8977**	breast	Plus2	22 (7C, 15N)	16	25
**22544**	breast	Plus2	18 (14C, 4N)	66	36
**41328**	colon	Plus2	20 (10C, 10N)	36	39
**14520**	liver	A2	43 (22C, 21N)	77	22
**14323**	liver	A2	66 (47C, 19N)	59	17
**10799**	lung	Plus2	19 (16C, 3N)	58	43
**14407**	ovarian	Plus2	24 (12C, 12N)	5	18
**3678**	thyroid	Plus2	14 (7C, 7N)	4	27
**6004**	thyroid	Plus2	18 (14C, 4N)	10	27

**Figure 1:**
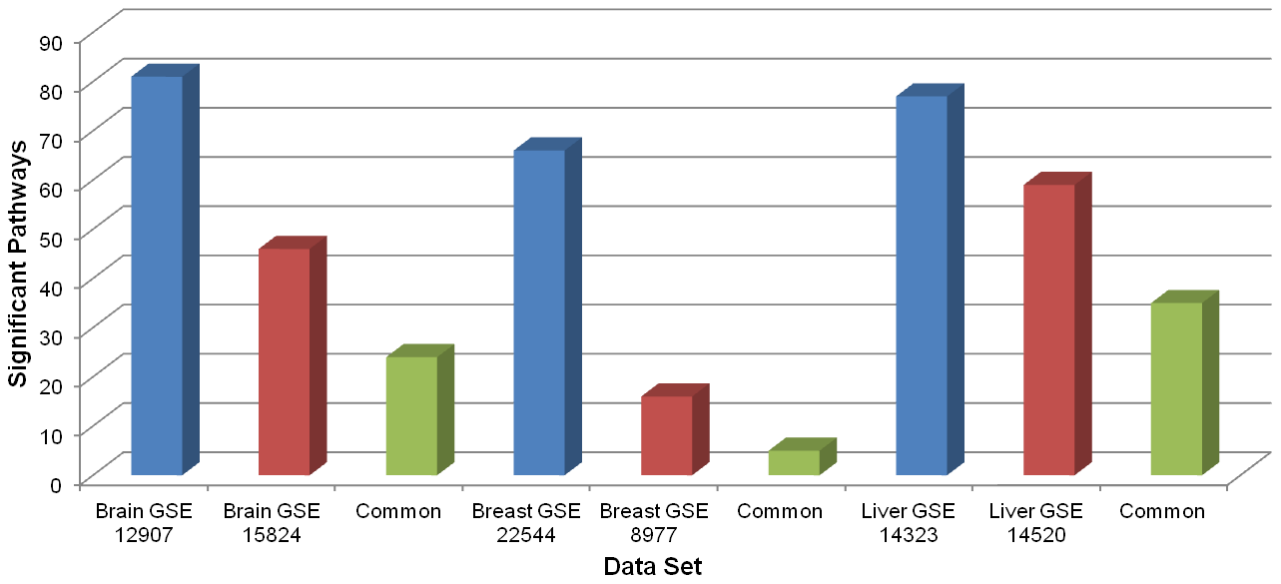
Commonality of significant pathways using the BPA analysis on the same cancer types.

We also investigated the commonality of significant pathways in cancer types represented by two data sets except for the thyroid cancer, which has resulted in very few significant pathways. These results for the BPA analysis are summarized in Figure 1. In the case of brain and liver cancer data sets, the common pathways consist of 52% and 59% of the dataset with the smaller number of pathways. In the breast cancer data sets, we see a lesser degree of agreement (∼31%). These commonalities are 60%, 41%, and 52% for the brain, breast, and liver datasets, respectively, using the SPIA analysis. However, SPIA uses a subset of the pathways investigated by the BPA system. When we consider only the pathways in the SPIA database, the commonalities in the BPA analysis are 73%, 45%, and 71% for the brain, breast, and liver datasets, respectively.

**Figure 2:**
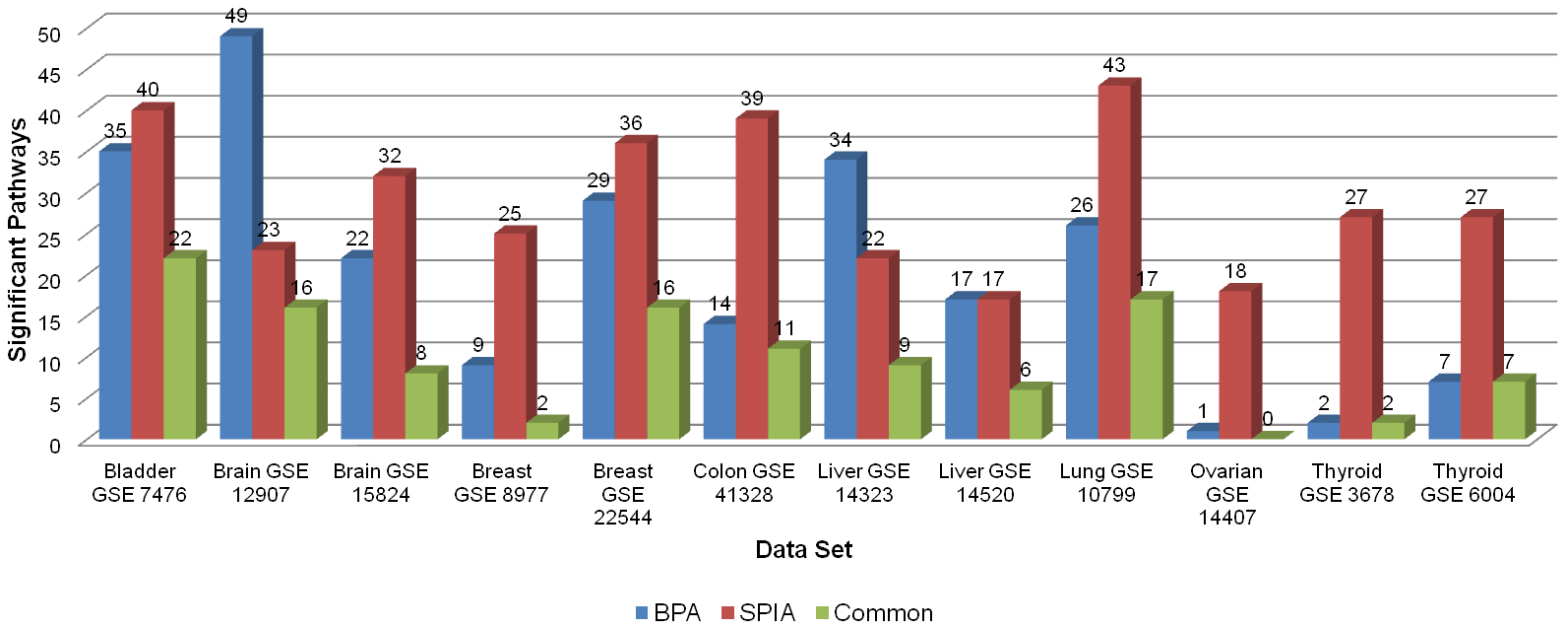
Number of pathways found significant in real microarray data sets using BPA and SPIA methods.

In Figure 2, we list the number of pathways identified by the two analysis methods when the pathway database is restricted to the one used by SPIA. On average, the number of pathways found to be significantly active by both methods is about 60% of the pathways of the algorithm with the smaller number of active pathways.

Although the improved BPA system outperformed the old BPA system on synthetic data sets (data not shown), we compared the performance of both methods on the real cancer microarray data. The list of pathways deemed significant by the old BPA system is represented in Table S4. The old BPA analysis revealed 127 pathways active in at least one of the cancer data sets and 18 of the pathways were found to be common to at least half of the data sets. In Table S5, we list the numbers of pathways identified as active by both BPA systems and indicate the number of pathways commonly identified by the two methods in each cancer data set.

These results on the real cancer data sets (Tables S4 and S5) indicate that the old BPA system fails to exhibit consistency for some of the datasets (e.g. 57 vs. 1 pathway identified by the new vs. old PBA in the “bladder” data set; 16 vs. 3 pathways identified by the new vs. old PBA in the “breast” data set; 58 vs. 0 pathway identified by the new vs. old PBA in the “lung” data set; and 10 vs. 0 pathway identified by the new vs. old PBA in the “thyroid” data set). We believe this is mainly due to the permutation test method introduced in the new BPA system where the old system fails to generate randomized data sets in pathways showing a constant fold change direction for its members (see Table 1). Some of the performance improvement can be attributed to the optimized discretization and scoring methods incorporated in the new BPA system. The old and new BPA sytems show, on average, a 28% overlap between the pathways identified in each data set. This level of agreement is significantly lower than the one observed between the new BPA and SPIA methods, which showed 60% overlap on average. Moreover, we obtained a 25% overlap on average between the old BPA and SPIA methods when the pathways identified for each real cancer microarray data set by the two methods were considered.

We also applied the improved BPA method on the NCI-60 cancer cell line microarray data set used in describing the Gene Set Enrichment Analysis (GSEA) method [31]. This data set contains microarray results (run on the Affymetrix HGU95Av2 platform) for 50 of the NCI-60 cell lines (www.broadinstitute.org/gsea/datasets.jsp). We used this data set to identify pathways deregulated following a mutation in the tumor suppressor p53 gene. Out of the 50 samples, 17 are wild type and 33 carry mutations in the p53 gene. The pathways identified as active by BPA due to the mutations in p53 are listed in Table 4.

**Table 4:** Significant pathways identified by the improved BPA system using the NCI-60 microarray data set on samples with and without p53 mutation (p53^+^: 17 Samples, p53^−^: 33 samples).

ID	Name
**hsa00650**	Butanoate metabolism
**hsa00061**	Fatty acid biosynthesis
**hsa05214**	Glioma
**hsa04916**	Melanogenesis
**hsa05218**	Melanoma
**hsa05212**	Pancreatic cancer
**hsa00230**	Purine metabolism
**hsa00240**	Pyrimidine metabolism
**hsa04660**	T cell receptor signaling pathway

## Discussion

Our synthetic data simulations identified k-means clustering as the best performing discretization method. We find this result reasonable as k-means uses the distribution in the data to minimize the total mean squared error with respect to the discretized values and the real FC occurrences. Also based on the synthetic data results, the scoring method that yielded the highest accuracy was the factorized normalized maximum likelihood (fNML) score [23]. This result was also expected as it has been shown that the BDe scoring scheme is very sensitive to the choice of prior hyper-parameters and AIC and BIC require some manual parameter setting and do not work well with small data sets, which is occasionally the case with HTBD [32]. fNML on the other hand is an information theory based optimized scoring method that has no tunable parameters.

In the real microarray data analysis using BPA, the pathway that came out in most of the cancer data sets as significantly active (8/12) is the Cell adhesion molecules (CAMs) pathway. CAMs are located on the cell surface and participate in the activity of a cell binding with other cells. One of the primary features of cancer cells is uncontrolled growth where the cells are immune to density-dependent inhibition. Cancer cells keep on growing, forming multiple levels, even when the cell density is increased. This is mainly due to the malfunctioning in CAMs, which has been shown to play an important role in cancer progression [33] and disrupting important signal-transduction pathways [34]. Specifically, CAMs have been shown to be involved in brain [35], bladder [36], breast [37], liver [38], lung [39] and thyroid [40] cancer; the cancer data sets where the proposed system found the CAM pathway as significantly activated.

Other pathways that need to be emphasized are “Citrate (TCA/tricarboxylic acid) cycle”, “Complement and coagulation cascade” and “Adipocytokine signaling” pathways that are found to be significantly active in 7 cancer data sets out of 12. Citrate cycle, also known as the tricarboxylic acid cycle (TCA cycle) or the Krebs cycle, is part of cellular respiration. It is a series of chemical reactions used by all aerobic organisms to generate energy. Its central importance to many biochemical pathways suggests that it was one of the earliest parts of cellular metabolism to evolve [41]. A recent study identified this cycle as a cancer-specific metabolic pathway [42]. In a wide range of tumor cells including the types included in our datasets, it is found that a mutation causes this cycle to run in reverse. Complement and coagulation cascade pathway can be explained in two parts: the complement system is a proteolytic cascade in blood plasma and a mediator of innate immunity, a nonspecific defense mechanism against pathogens, and blood coagulation is another series of proenzyme-to-serine protease conversions. This pathway is identified as significant for breast and liver cancer types in a functional cancer map, which has been established following the analysis of functional expression profiles of significantly enriched KEGG pathways across different tumor entities assigned to various tumor classes [43]. Adipocytokine signaling pathway is positively correlated with leptin production, which is an important regulator of energy intake and metabolic rate. Leptin and adiponectin are the most abundant adipocytokines and the best-studied molecules in this class so far. Recent tumor biological findings on the role of the most prominent adipocytokines leptin and adiponectin, which are involved in tumor growth, invasion and metastasis, show the effects of adipocytokines to brain and breast cancers [44], the types of cancer datasets where the BPA system found this pathway as significantly activated. There have been other additional studies that have shown the relation of adipocytokine signaling pathway to lung and liver cancers [59], [60].

Our synthetic data results show that the improved BPA system identifies the activity of a pathway with over 98% accuracy. Although there is no gold standart in assessing the active pathways regarding the real microarray data of a certain phenotype, BPA's reproducibility in the same cancer types has been over 50% in average. When the pathway database is limited to the one used by SPIA, this reproducibility exceeds 70%. Finally, when all the cancer datasets are considered, the agreement between the two methods is around 60%. Given the technical and biological variation, such a high degree of overlap between different pathway analysis schemes is very promising.

In an attempt to identify patways specific to particular cancer types, we investigated pathways that are consistently found to be active for the same cancer types (and non-active for the other cancer types) by the current BPA system. For brain cancer, “Parkinson's disease pathway (hsa05012)” was found active in both brain cancer data sets and only in one of the remaining 10 cancer data sets. Parkinson's disease (PD) is one of the most common neurodegeneretive disorders associated with cell loss in the substantia nigra region of the midbrain [45]. Recently, there have been studies that link the molecular mechanisms and genetic dispositions of the disease to cancer. Mutations in PARK2, one of the most common causes of early-onset PD, has been shown to play a central role in glioblastomas [46] exhibiting changes in almost identical residues in both the PD and the brain cancer samples. Identification of this pathway as active almost uniquely and consistently in brain cancer data sets implies that BPA is able to identify biologically meaningful pathways based on the underlying HTBD. In the liver cancer data sets, “Biotin metabolism (hsa00641)” and “3-Chloroacrylic acid degradation (hsa00780)” pathways were found to be active only in the two liver data sets. Biotin concentration is known to be high in cancerous tumors compared to normal tissues and biotin and other water-soluable vitamin B metabolisms have been shown to be important in hepatocellular carcinoma [47], [48]. Similarly, 3-Chloroacrylic acid degradation pathway has been shown to be active in hepatocellular carcinoma [49], [50].

Comparison of the old BPA system with the improved one proposed in this paper on the real cancer microarray data sets revealed that the old BPA system fails to identify pathways for some data sets and provides less biologically meaningful pathways showing insignificant agreement with the improved BPA and SPIA methods. Moreover, we see a less degree of agreement for the same cancer data sets in the old BPA system. For example, the old system identified only 3 pathways in one of the “brain” data sets compared to 122 pathways identified for another data set for the same cancer type. These differences are very likely due to the improved randomization, discretization, and scoring strategies introduced in the current BPA system.

Pathways that have been identified as significantly activated by BPA due to a p53 mutation are listed in Table 4. p53 is known to be involved in “melanogenesis” (hsa04916) through skin hyperpigmentation due to its role in cytokine receptor signaling [51], where alterations in p53 levels significantly affect the expression levels of melanogenic factors. p53 plays a central role in “melanoma” (hsa05218) as a therapeutic agent [52] and risk factor where certain signature hot-spot mutations in the p53 gene result in oncogenic transformation. Among the pathways listed in Table 4, p53 has been shown to be involved in “glioma” (hsa05214) [53] and “pancreatic cancer” (hsa5212) [54], [55]. Recently, it has been shown that antigen specific (CD4+) T cell response is critically affected by deregulation of p53 through “T cell receptor signaling pathway” (hsa04660) [56]. p53 is also known to be involved in “purine metabolism” (hsa00230) [57], “pyrimidine metabolism” (hsa00240) [58], and “fatty acid biosynthesis” (hsa00061) [59], [60]; other pathways identified as active by BPA due to mutations in p53. Overall, BPA was clearly able to identify pathways related to p53 using related microarray data. We believe that utilized synthetic, real, and benchmark data along with synthetic data sets render results that show the utility of the improved BPA system as a new resource for pathway analysis of HTBD.

## Supporting Information

Table S1 Prediction accuracy of different discretization methods on synthetic datasets. The superscripts denote the number of levels used in the discretization method.
(DOCX)

Table S2 Lists of active pathways identified by BPA on real cancer microarray data sets.
(DOCX)

Table S3 Lists of active pathways identified by SPIA on real cancer microarray data sets.
(DOCX)

Table S4 Lists of active pathways identified by the old BPA on real cancer microarray data sets.
(DOCX)

Table S5 Overlap of active pathways identified by the new and old BPA systemson real cancer microarray data sets.
(DOCX)
