# Supplementary material for: Bayesian Pathway Analysis of Cancer Microarray Data
Source: PLoS One. 2014 Jul 18;9(7):e102803. doi: 10.1371/journal.pone.0102803 (PMC4103872; doi:10.1371/journal.pone.0102803)
Supplement: Table S1 — Prediction accuracy of different discretization methods on synthetic datasets. The superscripts denote the number of levels used in the discretization method. (DOCX) [file pone.0102803.s001.docx]

**Table S1.** Prediction accuracy of different discretization methods on synthetic datasets. The superscripts denote the number of levels used in the discretization method.

|  | FC2^2^ | FC3^2^ | FC2^3^ | FC3^3^ | EWD^2^ | EWD^3^ | EWD^4^ | EWD^5^ | EFD^2^ | EFD^3^ | EFD^4^ | EFD^5^ | k-m^2^ | k-m^3^ |
| --- | --- | --- | --- | --- | --- | --- | --- | --- | --- | --- | --- | --- | --- | --- |
| D1 | 0.836 | 0.764 | 0.764 | 0.818 | 0.818 | 0.782 | 0.782 | 0.800 | 0.782 | 0.873 | 0.709 | 0.873 | 0.945 | 0.909 |
| D2 | 0.818 | 0.855 | 0.764 | 0.836 | 0.818 | 0.818 | 0.855 | 0.745 | 0.836 | 0.855 | 0.855 | 0.836 | 0.982 | 0.945 |
| D3 | 0.855 | 0.855 | 0.745 | 0.764 | 0.855 | 0.745 | 0.764 | 0.764 | 0.836 | 0.891 | 0.855 | 0.873 | 0.982 | 0.927 |
| D4 | 0.855 | 0.855 | 0.745 | 0.764 | 0.855 | 0.745 | 0.764 | 0.764 | 0.855 | 0.818 | 0.836 | 0.945 | 0.964 | 1.000 |
| D5 | 0.855 | 0.855 | 0.745 | 0.764 | 0.855 | 0.727 | 0.764 | 0.745 | 0.873 | 0.836 | 0.836 | 0.909 | 0.945 | 0.964 |
| D6 | 0.855 | 0.855 | 0.745 | 0.745 | 0.855 | 0.836 | 0.727 | 0.745 | 0.873 | 0.800 | 0.800 | 0.873 | 0.945 | 0.945 |
| D7 | 0.855 | 0.855 | 0.745 | 0.745 | 0.855 | 0.745 | 0.764 | 0.764 | 0.855 | 0.836 | 0.873 | 0.855 | 0.982 | 0.927 |
| D8 | 0.855 | 0.855 | 0.745 | 0.745 | 0.855 | 0.745 | 0.745 | 0.745 | 0.836 | 0.818 | 0.836 | 0.818 | 1.000 | 0.945 |
| D9 | 0.855 | 0.855 | 0.727 | 0.745 | 0.855 | 0.855 | 0.745 | 0.764 | 0.836 | 0.873 | 0.927 | 0.818 | 0.982 | 0.964 |
| D10 | 0.855 | 0.745 | 0.745 | 0.745 | 0.855 | 0.745 | 0.764 | 0.764 | 0.836 | 0.891 | 0.800 | 0.891 | 0.891 | 1.000 |
| **Avg** | 0.849 | 0.835 | 0.747 | 0.767 | 0.847 | 0.775 | 0.767 | 0.760 | 0.842 | 0.849 | 0.833 | 0.869 | **0.962** | 0.953 |
| **SD** | 0.012 | 0.042 | 0.010 | 0.033 | 0.015 | 0.046 | 0.034 | 0.017 | 0.026 | 0.032 | 0.057 | 0.040 | **0.031** | 0.030 |

|  | k-m^4^ | k-m^5^ | ck-m^2^ | ck-m^3^ | ck-m^4^ | ck-m^5^ | bk-m^2^ | k-m^4^ | k-m^5^ | ck-m^2^ | bk-m^3^ | bk-m^4^ | bk-m^5^ | ATG | ATL |
| --- | --- | --- | --- | --- | --- | --- | --- | --- | --- | --- | --- | --- | --- | --- | --- |
| D1 | 0.927 | 0.909 | 0.636 | 0.636 | 0.636 | 0.636 | 0.636 | 0.927 | 0.909 | 0.636 | 0.636 | 0.636 | 0.600 | 0.873 | 0.636 |
| D2 | 0.909 | 0.945 | 0.636 | 0.636 | 0.636 | 0.636 | 0.636 | 0.909 | 0.945 | 0.636 | 0.600 | 0.636 | 0.618 | 0.782 | 0.636 |
| D3 | 0.982 | 0.873 | 0.636 | 0.636 | 0.636 | 0.636 | 0.618 | 0.982 | 0.873 | 0.636 | 0.636 | 0.636 | 0.636 | 0.764 | 0.636 |
| D4 | 0.964 | 0.891 | 0.636 | 0.636 | 0.636 | 0.636 | 0.636 | 0.964 | 0.891 | 0.636 | 0.636 | 0.618 | 0.564 | 0.764 | 0.636 |
| D5 | 0.964 | 0.909 | 0.636 | 0.636 | 0.636 | 0.636 | 0.636 | 0.964 | 0.909 | 0.636 | 0.636 | 0.636 | 0.636 | 0.764 | 0.636 |
| D6 | 0.927 | 0.945 | 0.636 | 0.636 | 0.636 | 0.636 | 0.636 | 0.927 | 0.945 | 0.636 | 0.636 | 0.636 | 0.636 | 0.764 | 0.636 |
| D7 | 0.964 | 0.909 | 0.636 | 0.636 | 0.636 | 0.636 | 0.636 | 0.964 | 0.909 | 0.636 | 0.636 | 0.636 | 0.636 | 0.764 | 0.636 |
| D8 | 0.982 | 0.891 | 0.636 | 0.636 | 0.630 | 0.636 | 0.636 | 0.982 | 0.891 | 0.636 | 0.636 | 0.636 | 0.618 | 0.764 | 0.636 |
| D9 | 0.945 | 0.964 | 0.636 | 0.636 | 0.630 | 0.636 | 0.636 | 0.945 | 0.964 | 0.636 | 0.636 | 0.636 | 0.636 | 0.764 | 0.636 |
| D10 | 0.964 | 0.909 | 0.636 | 0.618 | 0.636 | 0.636 | 0.636 | 0.964 | 0.909 | 0.636 | 0.636 | 0.636 | 0.618 | 0.800 | 0.636 |
| Avg | 0.953 | 0.915 | 0.636 | 0.635 | 0.635 | 0.636 | 0.635 | 0.953 | 0.915 | 0.636 | 0.633 | 0.635 | 0.620 | 0.780 | 0.636 |
| SD | 0.025 | 0.028 | 0.000 | 0.006 | 0.003 | 0.000 | 0.006 | 0.025 | 0.028 | 0.000 | 0.011 | 0.006 | 0.023 | 0.035 | 0.000 |

*FC2: Fold Change cut-off 2; FC2: Fold Change cut-off 3; EWD: Equal Width Distribution; EFD: Equal Frequency Distribution; k-m: k-means; ck-m: column k-means; bk-m: bik-means; ATG: Automatic Threshold Global; ATL: Automatic Threshold Local; Avg: Average; SD: Standard Deviation. According to the simulation results, the best discretization method is the 2-level k-means discretization applied to the rows of the observation matrix (indicated in bold, underlined face with grey background).*
