# Supplementary material for: Bayesian Pathway Analysis of Cancer Microarray Data
Source: PLoS One. 2014 Jul 18;9(7):e102803. doi: 10.1371/journal.pone.0102803 (PMC4103872; doi:10.1371/journal.pone.0102803)
Supplement: Table S2 — Lists of active pathways identified by BPA on real cancer microarray data sets. (DOCX) [file pone.0102803.s002.docx]

**Table S2.** Lists of active pathways identified by BPA on real cancer microarray data sets.

| Cancer Type and GEO Number | | bladder | brain | brain | breast | breast | colon | liver | liver | lung | ovarian | thyroid | thyroid |  |
| --- | --- | --- | --- | --- | --- | --- | --- | --- | --- | --- | --- | --- | --- | --- |
| Pathway ID and Name | | GSE 7476 | GSE 12907 | GSE 15824 | GSE 8977 | GSE 22544 | GSE 41328 | GSE 14323 | GSE 14520 | GSE 100799 | GSE 14407 | GSE 3678 | GSE 6004 | Total |
| 04514 | Cell adhesion molecules (CAMs) | X | X | X |  | X |  | X | X | X |  | X |  | 8 |
| 00020 | Citrate cycle (TCA cycle) |  | X | X |  | X | X | X | X |  |  |  | X | 7 |
| 04610 | Complement and coagulation cascades | X | X |  |  | X | X | X | X | X |  |  |  | 7 |
| 04920 | Adipocytokine signaling pathway |  | X | X | X | X |  | X | X | X |  |  |  | 7 |
| 00100 | Biosynthesis of steroids | X |  |  |  | X | X | X | X | X |  |  |  | 6 |
| 00252 | Alanine and aspartate metabolism |  | X | X |  | X | X | X | X |  |  |  |  | 6 |
| 00272 | Cysteine metabolism | X |  |  | X | X |  |  | X | X | X |  |  | 6 |
| 00280 | Valine, leucine and isoleucine degradation | X | X |  |  | X | X | X | X |  |  |  |  | 6 |
| 00512 | O-Glycan biosynthesis |  | X | X |  |  | X | X | X | X |  |  |  | 6 |
| 00982 | Drug metabolism - cytochrome P450 |  |  | X |  | X | X | X | X | X |  |  |  | 6 |
| 04530 | Tight junction | X | X |  |  | X | X | X |  | X |  |  |  | 6 |
| 04662 | B cell receptor signaling pathway | X |  | X | X | X |  | X | X |  |  |  |  | 6 |
| 04664 | Fc epsilon RI signaling pathway | X | X | X |  |  |  | X | X | X |  |  |  | 6 |
| 04670 | Leukocyte transendothelial migration | X | X | X |  | X |  | X |  | X |  |  |  | 6 |
| 05222 | Small cell lung cancer | X | X | X |  | X |  | X |  |  |  |  | X | 6 |
| 00230 | Purine metabolism |  | X | X |  |  |  | X | X | X |  |  |  | 5 |
| 00240 | Pyrimidine metabolism |  |  | X |  | X | X | X | X |  |  |  |  | 5 |
| 00260 | Glycine, serine and threonine metabolism |  | X | X |  | X | X | X |  |  |  |  |  | 5 |
| 00511 | N-Glycan degradation | X |  | X |  | X |  | X | X |  |  |  |  | 5 |
| 00563 | Glycosylphosphatidylinositol(GPI)-anchor biosynthesis |  | X | X |  | X | X |  |  | X |  |  |  | 5 |
| 00590 | Arachidonic acid metabolism | X | X |  |  | X |  |  | X | X |  |  |  | 5 |
| 03320 | PPAR signaling pathway |  |  |  |  | X | X |  | X | X |  |  | X | 5 |
| 04010 | MAPK signaling pathway | X | X |  |  | X | X |  |  | X |  |  |  | 5 |
| 04115 | p53 signaling pathway | X | X |  |  | X |  |  | X | X |  |  |  | 5 |
| 04350 | TGF-beta signaling pathway | X | X |  |  | X |  | X |  | X |  |  |  | 5 |
| 04512 | ECM-receptor interaction | X | X |  |  | X | X |  | X |  |  |  |  | 5 |
| 04650 | Natural killer cell mediated cytotoxicity | X | X |  |  | X |  | X | X |  |  |  |  | 5 |
| 04710 | Circadian rhythm |  | X | X |  | X |  | X | X |  |  |  |  | 5 |
| 04720 | Long-term potentiation |  | X | X |  | X |  |  | X | X |  |  |  | 5 |
| 04742 | Taste transduction | X | X | X | X |  |  | X |  |  |  |  |  | 5 |
| 04910 | Insulin signaling pathway | X | X |  |  | X |  | X |  | X |  |  |  | 5 |
| 04916 | Melanogenesis | X | X | X |  | X |  |  |  | X |  |  |  | 5 |
| 05120 | Epithelial cell signaling in Helicobacter pylori infection | X | X | X |  |  | X |  |  | X |  |  |  | 5 |
| 05210 | Colorectal cancer | X | X |  |  |  | X | X |  |  |  |  | X | 5 |
| 05212 | Pancreatic cancer | X | X | X |  |  | X |  |  | X |  |  |  | 5 |
| 00040 | Pentose and glucuronate interconversions |  |  |  |  | X |  | X | X | X |  |  |  | 4 |
| 00071 | Fatty acid metabolism |  |  |  |  | X |  | X | X | X |  |  |  | 4 |
| 00220 | Urea cycle and metabolism of amino groups |  |  | X |  |  | X | X | X |  |  |  |  | 4 |
| 00330 | Arginine and proline metabolism | X | X |  | X |  | X |  |  |  |  |  |  | 4 |
| 00430 | Taurine and hypotaurine metabolism | X |  |  |  | X |  |  | X | X |  |  |  | 4 |
| 00520 | Nucleotide sugars metabolism |  | X |  |  | X |  | X |  | X |  |  |  | 4 |
| 00532 | Chondroitin sulfate biosynthesis |  | X |  |  |  |  | X | X | X |  |  |  | 4 |
| 00604 | Glycosphingolipid biosynthesis - ganglio series | X | X |  |  |  |  |  | X | X |  |  |  | 4 |
| 00640 | Propanoate metabolism | X | X |  |  |  |  | X |  | X |  |  |  | 4 |
| 00750 | Vitamin B6 metabolism | X |  |  |  | X |  |  |  | X |  |  | X | 4 |
| 00760 | Nicotinate and nicotinamide metabolism |  | X |  |  | X |  |  | X | X |  |  |  | 4 |
| 04020 | Calcium signaling pathway | X | X |  |  |  |  | X |  |  |  | X |  | 4 |
| 04310 | Wnt signaling pathway |  |  |  |  |  | X | X | X | X |  |  |  | 4 |
| 04360 | Axon guidance | X | X |  |  | X |  |  |  | X |  |  |  | 4 |
| 04612 | Antigen processing and presentation | X | X |  |  |  |  | X | X |  |  |  |  | 4 |
| 04740 | Olfactory transduction |  | X |  |  | X |  | X | X |  |  |  |  | 4 |
| 04912 | GnRH signaling pathway |  | X | X | X |  |  | X |  |  |  |  |  | 4 |
| 04930 | Type II diabetes mellitus |  |  | X | X | X |  | X |  |  |  |  |  | 4 |
| 05010 | Alzheimer's disease |  | X |  |  | X |  | X | X |  |  |  |  | 4 |
| 05014 | Amyotrophic lateral sclerosis (ALS) |  | X |  | X |  |  | X | X |  |  |  |  | 4 |
| 05130 | Pathogenic Escherichia coli infection - EHEC |  |  |  | X | X | X | X |  |  |  |  |  | 4 |
| 05131 | Pathogenic Escherichia coli infection - EPEC | X |  |  |  | X | X | X |  |  |  |  |  | 4 |
| 05220 | Chronic myeloid leukemia | X | X | X |  |  |  |  |  | X |  |  |  | 4 |
| 05223 | Non-small cell lung cancer | X | X |  |  |  |  | X |  |  |  |  | X | 4 |
| 00010 | Glycolysis / Gluconeogenesis |  |  |  |  | X |  |  | X | X |  |  |  | 3 |
| 00030 | Pentose phosphate pathway |  |  | X |  |  |  |  | X | X |  |  |  | 3 |
| 00052 | Galactose metabolism |  |  | X |  | X |  |  | X |  |  |  |  | 3 |
| 00061 | Fatty acid biosynthesis | X | X |  |  |  |  | X |  |  |  |  |  | 3 |
| 00062 | Fatty acid elongation in mitochondria |  |  |  |  |  | X |  | X | X |  |  |  | 3 |
| 00251 | Glutamate metabolism |  |  |  |  | X |  | X | X |  |  |  |  | 3 |
| 00271 | Methionine metabolism |  | X |  |  |  |  | X | X |  |  |  |  | 3 |
| 00300 | Lysine biosynthesis | X | X |  |  |  |  | X |  |  |  |  |  | 3 |
| 00340 | Histidine metabolism |  |  | X |  |  |  | X | X |  |  |  |  | 3 |
| 00510 | N-Glycan biosynthesis | X |  |  |  | X |  |  |  | X |  |  |  | 3 |
| 00561 | Glycerolipid metabolism | X |  |  |  | X |  |  |  | X |  |  |  | 3 |
| 00564 | Glycerophospholipid metabolism |  | X |  |  |  |  | X |  | X |  |  |  | 3 |
| 00592 | alpha-Linolenic acid metabolism |  |  |  | X |  |  | X | X |  |  |  |  | 3 |
| 00600 | Sphingolipid metabolism | X |  |  |  | X |  | X |  |  |  |  |  | 3 |
| 00620 | Pyruvate metabolism |  | X |  |  | X |  | X |  |  |  |  |  | 3 |
| 00670 | One carbon pool by folate | X |  |  |  | X | X |  |  |  |  |  |  | 3 |
| 00680 | Methane metabolism |  | X |  |  |  |  |  | X | X |  |  |  | 3 |
| 00920 | Sulfur metabolism |  |  | X |  |  |  | X | X |  |  |  |  | 3 |
| 01040 | Biosynthesis of unsaturated fatty acids |  |  |  |  |  |  | X | X |  |  | X |  | 3 |
| 03050 | Proteasome |  | X | X |  |  |  |  | X |  |  |  |  | 3 |
| 03060 | Protein export |  |  |  |  |  |  | X | X |  | X |  |  | 3 |
| 04012 | ErbB signaling pathway | X | X |  |  | X |  |  |  |  |  |  |  | 3 |
| 04110 | Cell cycle | X |  |  |  |  | X |  |  | X |  |  |  | 3 |
| 04210 | Apoptosis | X | X |  |  |  |  |  |  | X |  |  |  | 3 |
| 04370 | VEGF signaling pathway | X |  | X |  |  |  | X |  |  |  |  |  | 3 |
| 04510 | Focal adhesion | X | X |  |  |  |  |  |  | X |  |  |  | 3 |
| 04620 | Toll-like receptor signaling pathway |  |  | X |  |  | X |  |  | X |  |  |  | 3 |
| 04660 | T cell receptor signaling pathway |  | X |  |  | X |  |  |  | X |  |  |  | 3 |
| 04730 | Long-term depression |  | X |  |  |  |  | X |  |  | X |  |  | 3 |
| 05012 | Parkinson's disease |  | X | X |  |  |  | X |  |  |  |  |  | 3 |
| 05110 | Vibrio cholerae infection |  |  |  |  | X |  | X |  | X |  |  |  | 3 |
| 05213 | Endometrial cancer | X | X | X |  |  |  |  |  |  |  |  |  | 3 |
| 05214 | Glioma |  | X |  |  | X |  |  |  |  |  |  | X | 3 |
| 05215 | Prostate cancer | X | X |  |  |  |  |  | X |  |  |  |  | 3 |
| 05216 | Thyroid cancer |  | X |  |  |  | X |  |  |  |  |  | X | 3 |
| 05218 | Melanoma | X | X |  |  |  |  |  |  |  |  |  | X | 3 |
| 05310 | Asthma |  | X |  | X |  |  | X |  |  |  |  |  | 3 |
| 05320 | Autoimmune thyroid disease |  |  | X |  |  |  | X | X |  |  |  |  | 3 |
| 05330 | Allograft rejection |  | X | X |  |  |  | X |  |  |  |  |  | 3 |
| 00051 | Fructose and mannose metabolism |  |  |  |  | X |  |  | X |  |  |  |  | 2 |
| 00053 | Ascorbate and aldarate metabolism |  | X |  |  |  |  | X |  |  |  |  |  | 2 |
| 00232 | Caffeine metabolism |  |  |  | X |  |  |  |  |  | X |  |  | 2 |
| 00363 | Bisphenol A degradation | X |  |  |  | X |  |  |  |  |  |  |  | 2 |
| 00440 | Aminophosphonate metabolism |  |  |  |  |  |  | X |  | X |  |  |  | 2 |
| 00500 | Starch and sucrose metabolism |  |  |  |  | X |  |  |  | X |  |  |  | 2 |
| 00562 | Inositol phosphate metabolism |  | X |  |  |  | X |  |  |  |  |  |  | 2 |
| 00565 | Ether lipid metabolism |  |  |  |  | X |  |  | X |  |  |  |  | 2 |
| 00601 | Glycosphingolipid biosynthesis - lacto and neolacto series | X |  |  |  |  |  |  |  | X |  |  |  | 2 |
| 00603 | Glycosphingolipid biosynthesis - globo series |  |  | X |  |  | X |  |  |  |  |  |  | 2 |
| 00641 | 3-Chloroacrylic acid degradation |  |  |  |  |  |  | X | X |  |  |  |  | 2 |
| 00720 | Reductive carboxylate cycle (CO2 fixation) |  | X |  |  | X |  |  |  |  |  |  |  | 2 |
| 00780 | Biotin metabolism |  |  |  |  |  |  | X | X |  |  |  |  | 2 |
| 00830 | Retinol metabolism |  | X |  |  | X |  |  |  |  |  |  |  | 2 |
| 00900 | Terpenoid biosynthesis |  |  |  |  | X | X |  |  |  |  |  |  | 2 |
| 00910 | Nitrogen metabolism |  | X |  |  |  |  |  | X |  |  |  |  | 2 |
| 00960 | Alkaloid biosynthesis II |  | X |  |  | X |  |  |  |  |  |  |  | 2 |
| 00970 | Aminoacyl-tRNA biosynthesis |  |  | X | X |  |  |  |  |  |  |  |  | 2 |
| 00980 | Metabolism of xenobiotics by cytochrome P450 | X |  |  |  |  | X |  |  |  |  |  |  | 2 |
| 00983 | Drug metabolism - other enzymes |  |  |  |  |  | X | X |  |  |  |  |  | 2 |
| 03020 | RNA polymerase |  |  | X |  |  |  |  | X |  |  |  |  | 2 |
| 04070 | Phosphatidylinositol signaling system |  | X |  |  | X |  |  |  |  |  |  |  | 2 |
| 04080 | Neuroactive ligand-receptor interaction |  |  | X | X |  |  |  |  |  |  |  |  | 2 |
| 04140 | Regulation of autophagy | X |  |  |  |  |  |  |  | X |  |  |  | 2 |
| 04150 | mTOR signaling pathway |  | X | X |  |  |  |  |  |  |  |  |  | 2 |
| 04330 | Notch signaling pathway |  | X |  |  | X |  |  |  |  |  |  |  | 2 |
| 04520 | Adherens junction |  | X |  |  |  |  |  |  | X |  |  |  | 2 |
| 04630 | Jak-STAT signaling pathway | X | X |  |  |  |  |  |  |  |  |  |  | 2 |
| 04640 | Hematopoietic cell lineage |  |  | X |  |  |  |  | X |  |  |  |  | 2 |
| 04950 | Maturity onset diabetes of the young |  | X |  |  |  |  | X |  |  |  |  |  | 2 |
| 05211 | Renal cell carcinoma |  |  |  |  |  |  | X |  | X |  |  |  | 2 |
| 05217 | Basal cell carcinoma |  | X |  |  |  |  |  |  | X |  |  |  | 2 |
| 05221 | Acute myeloid leukemia | X | X |  |  |  |  |  |  |  |  |  |  | 2 |
| 05340 | Primary immunodeficiency |  |  |  | X |  |  | X |  |  |  |  |  | 2 |
| 00072 | Synthesis and degradation of ketone bodies |  |  |  |  |  |  |  | X |  |  |  |  | 1 |
| 00120 | Bile acid biosynthesis |  |  |  |  |  | X |  |  |  |  |  |  | 1 |
| 00130 | Ubiquinone and menaquinone biosynthesis |  |  |  |  | X |  |  |  |  |  |  |  | 1 |
| 00140 | C21-Steroid hormone metabolism |  |  |  |  |  |  |  | X |  |  |  |  | 1 |
| 00190 | Oxidative phosphorylation |  | X |  |  |  |  |  |  |  |  |  |  | 1 |
| 00290 | Valine, leucine and isoleucine biosynthesis |  | X |  |  |  |  |  |  |  |  |  |  | 1 |
| 00350 | Tyrosine metabolism |  |  |  | X |  |  |  |  |  |  |  |  | 1 |
| 00360 | Phenylalanine metabolism |  |  |  |  | X |  |  |  |  |  |  |  | 1 |
| 00380 | Tryptophan metabolism | X |  |  |  |  |  |  |  |  |  |  |  | 1 |
| 00410 | beta-Alanine metabolism |  |  |  |  |  |  |  |  | X |  |  |  | 1 |
| 00450 | Selenoamino acid metabolism |  |  |  |  |  |  | X |  |  |  |  |  | 1 |
| 00460 | Cyanoamino acid metabolism |  |  |  |  | X |  |  |  |  |  |  |  | 1 |
| 00471 | D-Glutamine and D-glutamate metabolism |  |  |  |  |  |  |  |  | X |  |  |  | 1 |
| 00480 | Glutathione metabolism |  |  |  |  |  |  | X |  |  |  |  |  | 1 |
| 00530 | Aminosugars metabolism |  |  | X |  |  |  |  |  |  |  |  |  | 1 |
| 00531 | Glycosaminoglycan degradation |  |  |  |  |  |  | X |  |  |  |  |  | 1 |
| 00533 | Keratan sulfate biosynthesis |  |  | X |  |  |  |  |  |  |  |  |  | 1 |
| 00534 | Heparan sulfate biosynthesis |  |  |  |  |  |  |  |  | X |  |  |  | 1 |
| 00630 | Glyoxylate and dicarboxylate metabolism |  |  |  |  |  | X |  |  |  |  |  |  | 1 |
| 00643 | Styrene degradation |  |  |  |  |  | X |  |  |  |  |  |  | 1 |
| 00650 | Butanoate metabolism |  |  |  |  |  | X |  |  |  |  |  |  | 1 |
| 00730 | Thiamine metabolism |  |  |  |  |  |  | X |  |  |  |  |  | 1 |
| 00770 | Pantothenate and CoA biosynthesis |  |  |  |  |  |  |  |  |  |  |  | X | 1 |
| 00790 | Folate biosynthesis |  |  |  |  |  |  |  |  |  | X |  |  | 1 |
| 00860 | Porphyrin and chlorophyll metabolism |  |  | X |  |  |  |  |  |  |  |  |  | 1 |
| 00930 | Caprolactam degradation |  |  |  |  |  |  | X |  |  |  |  |  | 1 |
| 00950 | Alkaloid biosynthesis I |  |  |  |  |  |  |  |  | X |  |  |  | 1 |
| 01030 | Glycan structures - biosynthesis 1 |  |  |  |  |  |  |  | X |  |  |  |  | 1 |
| 01032 | Glycan structures - degradation |  |  | X |  |  |  |  |  |  |  |  |  | 1 |
| 02010 | ABC transporters - General |  |  |  |  |  |  | X |  |  |  |  |  | 1 |
| 03410 | Base excision repair | X |  |  |  |  |  |  |  |  |  |  |  | 1 |
| 03420 | Nucleotide excision repair |  | X |  |  |  |  |  |  |  |  |  |  | 1 |
| 04060 | Cytokine-cytokine receptor interaction | X |  |  |  |  |  |  |  |  |  |  |  | 1 |
| 04130 | SNARE interactions in vesicular transport |  | X |  |  |  |  |  |  |  |  |  |  | 1 |
| 04614 | Renin-angiotensin system |  |  |  |  |  |  | X |  |  |  |  |  | 1 |
| 04810 | Regulation of actin cytoskeleton |  |  |  |  |  |  |  |  |  |  | X |  | 1 |
| 05050 | Dentatorubropallidoluysian atrophy (DRPLA) |  |  |  |  |  |  | X |  |  |  |  |  | 1 |
| 05219 | Bladder cancer |  |  |  |  | X |  |  |  |  |  |  |  | 1 |
| 05332 | Graft-versus-host disease |  |  |  |  |  |  | X |  |  |  |  |  | 1 |
| TOTAL | | 57 | 81 | 46 | 16 | 66 | 36 | 77 | 59 | 58 | 5 | 4 | 10 |  |
