# Supplementary material for: Bayesian Pathway Analysis of Cancer Microarray Data
Source: PLoS One. 2014 Jul 18;9(7):e102803. doi: 10.1371/journal.pone.0102803 (PMC4103872; doi:10.1371/journal.pone.0102803)
Supplement: Table S3 — Lists of active pathways identified by SPIA on real cancer microarray data sets. (DOCX) [file pone.0102803.s003.docx]

**Table S3.** Lists of active pathways identified by SPIA on real cancer microarray data sets.

| Cancer Type and GEO Number | | bladder | brain | brain | breast | breast | colon | liver | liver | lung | ovarian | thyroid | thyroid |  |
| --- | --- | --- | --- | --- | --- | --- | --- | --- | --- | --- | --- | --- | --- | --- |
| Pathway ID and Name | | GSE 7476 | GSE 12907 | GSE 15824 | GSE 8977 | GSE 22544 | GSE 41328 | GSE 14323 | GSE 14520 | GSE 10799 | GSE 14407 | GSE 3678 | GSE 6004 | Total |
| 04510 | Focal adhesion | X | X | X | X | X | X | X | X | X | X | X | X | 12 |
| 04810 | Regulation of actin cytoskeleton | X | X | X | X | X | X | X | X | X | X | X | X | 12 |
| 05222 | Small cell lung cancer | X | X | X | X | X | X | X | X | X | X | X | X | 12 |
| 04512 | ECM-receptor interaction | X | X | X |  | X | X | X | X | X | X | X | X | 11 |
| 05215 | Prostate cancer | X |  | X | X | X | X | X | X | X | X | X | X | 11 |
| 04360 | Axon guidance | X | X | X | X | X | X |  |  | X | X | X | X | 10 |
| 05218 | Melanoma | X |  | X | X | X | X | X |  | X | X | X | X | 10 |
| 04540 | Gap junction | X | X |  | X | X | X | X |  | X | X | X |  | 9 |
| 05212 | Pancreatic cancer | X | X | X |  | X | X |  |  | X | X | X | X | 9 |
| 04530 | Tight junction | X |  |  | X | X | X |  |  | X | X | X | X | 8 |
| 05211 | Renal cell carcinoma | X | X | X | X |  | X | X |  | X |  |  | X | 8 |
| 05214 | Glioma | X | X |  |  | X | X |  | X | X |  | X | X | 8 |
| 05220 | Chronic myeloid leukemia | X | X | X |  | X | X |  |  | X |  | X | X | 8 |
| 04010 | MAPK signaling pathway | X |  | X |  | X | X | X |  | X | X |  |  | 7 |
| 04012 | ErbB signaling pathway | X | X |  |  | X | X |  |  | X |  | X | X | 7 |
| 04060 | Cytokine-cytokine receptor interaction |  |  |  | X | X | X | X | X | X |  |  | X | 7 |
| 04110 | Cell cycle | X |  |  |  | X | X | X | X | X | X |  |  | 7 |
| 05210 | Colorectal cancer | X |  | X |  | X | X |  |  | X |  | X | X | 7 |
| 05213 | Endometrial cancer | X |  | X |  | X | X |  |  | X |  | X | X | 7 |
| 05223 | Non-small cell lung cancer | X |  |  |  | X | X |  | X | X |  | X | X | 7 |
| 03320 | PPAR signaling pathway |  |  |  |  | X | X |  | X | X |  | X | X | 6 |
| 04020 | Calcium signaling pathway | X |  |  |  | X | X | X |  | X |  | X |  | 6 |
| 04210 | Apoptosis | X | X | X |  |  | X |  |  | X |  | X |  | 6 |
| 04310 | Wnt signaling pathway | X |  | X |  |  | X |  |  | X | X | X |  | 6 |
| 04350 | TGF-beta signaling pathway | X |  | X |  |  | X |  |  | X | X | X |  | 6 |
| 04670 | Leukocyte transendothelial migration |  | X | X | X | X |  |  |  | X |  | X |  | 6 |
| 05130 | Pathogenic Escherichia coli infection | X | X | X | X |  |  |  | X | X |  |  |  | 6 |
| 05131 | Shigellosis | X |  | X | X |  |  |  | X | X | X |  |  | 6 |
| 05322 | Systemic lupus erythematosus |  | X | X | X | X |  |  | X | X |  |  |  | 6 |
| 04115 | p53 signaling pathway | X |  |  |  |  | X | X |  | X |  | X |  | 5 |
| 04370 | VEGF signaling pathway |  | X |  |  | X | X |  | X |  |  |  | X | 5 |
| 04610 | Complement and coagulation cascades |  |  | X |  | X | X |  | X |  | X |  |  | 5 |
| 04730 | Long-term depression | X | X |  |  |  | X |  |  | X |  | X |  | 5 |
| 05120 | Epithelial cell signaling in Helicobacter pylori infection |  |  |  | X | X | X |  |  | X | X |  |  | 5 |
| 04612 | Antigen processing and presentation |  | X | X | X |  |  | X |  |  |  |  |  | 4 |
| 04650 | Natural killer cell mediated cytotoxicity |  |  | X | X |  |  | X |  | X |  |  |  | 4 |
| 04662 | B cell receptor signaling pathway |  |  |  | X | X |  |  |  | X |  |  | X | 4 |
| 04664 | Fc epsilon RI signaling pathway |  |  | X | X |  |  | X |  | X |  |  |  | 4 |
| 04912 | GnRH signaling pathway | X |  |  |  | X | X | X |  |  |  |  |  | 4 |
| 04920 | Adipocytokine signaling pathway |  |  |  |  | X | X |  | X |  |  | X |  | 4 |
| 05010 | Alzheimer's disease | X | X |  |  |  | X |  |  | X |  |  |  | 4 |
| 05014 | Amyotrophic lateral sclerosis (ALS) | X | X | X |  |  |  |  | X |  |  |  |  | 4 |
| 05216 | Thyroid cancer |  |  |  |  | X | X |  |  | X |  |  | X | 4 |
| 05219 | Bladder cancer | X |  |  |  |  | X | X |  |  |  |  | X | 4 |
| 05221 | Acute myeloid leukemia | X |  |  |  | X | X |  |  | X |  |  |  | 4 |
| 04070 | Phosphatidylinositol signaling system | X | X |  |  |  | X |  |  |  |  |  |  | 3 |
| 04150 | mTOR signaling pathway |  |  |  |  | X |  |  |  | X |  |  | X | 3 |
| 04620 | Toll-like receptor signaling pathway |  |  | X | X |  |  |  |  | X |  |  |  | 3 |
| 04660 | T cell receptor signaling pathway |  |  |  |  | X |  | X |  |  |  |  | X | 3 |
| 04710 | Circadian rhythm - mammal | X |  |  | X |  |  |  |  |  |  | X |  | 3 |
| 04910 | Insulin signaling pathway | X |  |  |  | X |  |  |  |  |  |  | X | 3 |
| 04940 | Type I diabetes mellitus |  |  | X | X |  |  | X |  |  |  |  |  | 3 |
| 05016 | Huntington's disease | X |  |  |  |  | X |  |  | X |  |  |  | 3 |
| 05110 | Vibrio cholerae infection |  |  |  |  | X |  | X |  |  |  |  | X | 3 |
| 04330 | Notch signaling pathway |  |  | X |  |  |  |  |  | X |  |  |  | 2 |
| 04720 | Long-term potentiation | X | X |  |  |  |  |  |  |  |  |  |  | 2 |
| 04916 | Melanogenesis | X |  | X |  |  |  |  |  |  |  |  |  | 2 |
| 04930 | Type II diabetes mellitus |  |  |  |  | X |  |  |  | X |  |  |  | 2 |
| 05012 | Parkinson's disease | X |  |  |  |  | X |  |  |  |  |  |  | 2 |
| 05217 | Basal cell carcinoma | X |  | X |  |  |  |  |  |  |  |  |  | 2 |
| 05330 | Allograft rejection |  |  |  | X |  |  | X |  |  |  |  |  | 2 |
| 04080 | Neuroactive ligand-receptor interaction |  | X |  |  |  |  |  |  |  |  |  |  | 1 |
| 04130 | SNARE interactions in vesicular transport |  |  |  |  |  |  |  |  |  |  |  | X | 1 |
| 04140 | Regulation of autophagy |  |  |  |  |  |  |  |  |  | X |  |  | 1 |
| 04340 | Hedgehog signaling pathway |  |  | X |  |  |  |  |  |  |  |  |  | 1 |
| 04630 | Jak-STAT signaling pathway |  |  |  |  |  |  |  |  |  |  | X |  | 1 |
| 04740 | Olfactory transduction |  |  | X |  |  |  |  |  |  |  |  |  | 1 |
| 05320 | Autoimmune thyroid disease |  |  |  | X |  |  |  |  |  |  |  |  | 1 |
| 05332 | Graft-versus-host disease |  |  |  | X |  |  |  |  |  |  |  |  | 1 |
| TOTAL | | 40 | 23 | 32 | 25 | 36 | 39 | 22 | 17 | 43 | 18 | 27 | 27 |  |
