# Supplementary material for: Bayesian Pathway Analysis of Cancer Microarray Data
Source: PLoS One. 2014 Jul 18;9(7):e102803. doi: 10.1371/journal.pone.0102803 (PMC4103872; doi:10.1371/journal.pone.0102803)
Supplement: Table S4 — Lists of active pathways identified by the old BPA on real cancer microarray data sets. (DOCX) [file pone.0102803.s004.docx]

**Table S4.** Lists of active pathways identified by the old BPA on real cancer microarray data sets.

| Cancer Type and GEO Number | | bladder | brain | brain | breast | breast | colon | liver | liver | lung | ovarian | thyroid | thyroid |  |
| --- | --- | --- | --- | --- | --- | --- | --- | --- | --- | --- | --- | --- | --- | --- |
| Pathway ID and Name | | GSE 7476 | GSE 12907 | GSE 15824 | GSE 8977 | GSE 22544 | GSE 41328 | GSE 14323 | GSE 14520 | GSE 10799 | GSE 14407 | GSE 3678 | GSE 6004 | Total |
| hsa00020 | Citrate cycle (TCA cycle) |  | X | X |  | X | X | X | X |  | X |  |  | 7 |
| hsa00030 | Pentose phosphate pathway |  | X | X |  | X | X | X | X |  | X |  |  | 7 |
| hsa00280 | Valine, leucine and isoleucine degradation |  |  | X | X | X | X | X | X |  | X |  |  | 7 |
| hsa00480 | Glutathione metabolism |  | X | X |  | X | X | X | X |  | X |  |  | 7 |
| hsa00600 | Sphingolipid metabolism |  | X | X |  | X | X | X | X |  | X |  |  | 7 |
| hsa04070 | Phosphatidylinositol signaling system |  | X | X |  | X | X | X | X |  | X |  |  | 7 |
| hsa00010 | Glycolysis / Gluconeogenesis |  |  | X | X | X |  | X | X |  | X |  |  | 6 |
| hsa00052 | Galactose metabolism |  |  | X |  | X | X | X | X |  | X |  |  | 6 |
| hsa00100 | Biosynthesis of steroids |  | X | X |  | X | X | X | X |  |  |  |  | 6 |
| hsa00240 | Pyrimidine metabolism |  | X | X |  | X | X | X | X |  |  |  |  | 6 |
| hsa00251 | Glutamate metabolism |  | X | X |  | X | X | X | X |  |  |  |  | 6 |
| hsa00252 | Alanine and aspartate metabolism |  | X | X |  | X | X | X | X |  |  |  |  | 6 |
| hsa00590 | Arachidonic acid metabolism |  | X | X |  | X | X | X | X |  |  |  |  | 6 |
| hsa00604 | Glycosphingolipid biosynthesis - ganglio series |  | X | X |  | X | X | X | X |  |  |  |  | 6 |
| hsa04512 | ECM-receptor interaction |  |  | X |  | X | X | X | X |  | X |  |  | 6 |
| hsa04520 | Adherens junction |  |  | X |  | X | X | X | X |  | X |  |  | 6 |
| hsa04650 | Natural killer cell mediated cytotoxicity |  | X | X |  | X | X | X | X |  |  |  |  | 6 |
| hsa04662 | B cell receptor signaling pathway |  |  | X |  | X | X | X | X |  | X |  |  | 6 |
| hsa00062 | Fatty acid elongation in mitochondria |  |  |  |  | X | X | X | X |  | X |  |  | 5 |
| hsa00071 | Fatty acid metabolism |  |  |  |  | X | X | X | X |  | X |  |  | 5 |
| hsa00500 | Starch and sucrose metabolism |  |  | X |  | X | X | X | X |  |  |  |  | 5 |
| hsa00670 | One carbon pool by folate |  |  |  |  | X | X | X | X |  | X |  |  | 5 |
| hsa00760 | Nicotinate and nicotinamide metabolism |  |  | X |  | X | X | X | X |  |  |  |  | 5 |
| hsa00910 | Nitrogen metabolism |  |  | X |  | X | X | X | X |  |  |  |  | 5 |
| hsa00980 | Metabolism of xenobiotics by cytochrome P450 |  |  | X |  | X |  | X | X |  | X |  |  | 5 |
| hsa00983 | Drug metabolism - other enzymes |  |  | X |  | X | X | X | X |  |  |  |  | 5 |
| hsa04110 | Cell cycle |  |  | X |  | X |  | X | X |  | X |  |  | 5 |
| hsa04510 | Focal adhesion |  | X |  |  | X | X | X | X |  |  |  |  | 5 |
| hsa04514 | Cell adhesion molecules (CAMs) |  |  | X |  | X | X | X | X |  |  |  |  | 5 |
| hsa04710 | Circadian rhythm |  | X | X |  | X |  |  | X |  | X |  |  | 5 |
| hsa04720 | Long-term potentiation |  |  | X | X | X |  | X |  |  | X |  |  | 5 |
| hsa04930 | Type II diabetes mellitus |  |  | X |  | X | X | X | X |  |  |  |  | 5 |
| hsa00072 | Synthesis and degradation of ketone bodies |  |  |  |  | X | X |  | X |  | X |  |  | 4 |
| hsa00220 | Urea cycle and metabolism of amino groups |  | X |  |  | X |  | X | X |  |  |  |  | 4 |
| hsa00271 | Methionine metabolism |  |  |  |  | X | X | X | X |  |  |  |  | 4 |
| hsa00330 | Arginine and proline metabolism |  |  | X |  | X |  | X | X |  |  |  |  | 4 |
| hsa00510 | N-Glycan biosynthesis |  |  |  |  | X | X | X |  |  | X |  |  | 4 |
| hsa00562 | Inositol phosphate metabolism |  |  | X |  | X | X |  |  |  | X |  |  | 4 |
| hsa00620 | Pyruvate metabolism |  |  |  |  | X | X | X |  |  | X |  |  | 4 |
| hsa00640 | Propanoate metabolism |  |  |  |  | X | X | X | X |  |  |  |  | 4 |
| hsa00830 | Retinol metabolism |  |  |  |  | X | X | X | X |  |  |  |  | 4 |
| hsa00860 | Porphyrin and chlorophyll metabolism |  |  |  |  | X | X |  | X |  | X |  |  | 4 |
| hsa00982 | Drug metabolism - cytochrome P450 |  |  |  |  | X |  | X | X |  | X |  |  | 4 |
| hsa03320 | PPAR signaling pathway |  | X |  |  | X | X |  | X |  |  |  |  | 4 |
| hsa04150 | mTOR signaling pathway |  |  |  |  | X | X | X | X |  |  |  |  | 4 |
| hsa04350 | TGF-beta signaling pathway |  |  | X |  | X |  | X | X |  |  |  |  | 4 |
| hsa04610 | Complement and coagulation cascades |  |  | X |  | X |  | X | X |  |  |  |  | 4 |
| hsa04630 | Jak-STAT signaling pathway |  |  | X |  | X |  | X | X |  |  |  |  | 4 |
| hsa04660 | T cell receptor signaling pathway |  |  |  |  | X |  | X | X |  | X |  |  | 4 |
| hsa00230 | Purine metabolism |  |  |  |  | X | X | X |  |  |  |  |  | 3 |
| hsa00410 | beta-Alanine metabolism |  |  |  |  | X |  | X | X |  |  |  |  | 3 |
| hsa00512 | O-Glycan biosynthesis |  |  |  |  | X | X | X |  |  |  |  |  | 3 |
| hsa00630 | Glyoxylate and dicarboxylate metabolism |  |  |  |  | X |  | X | X |  |  |  |  | 3 |
| hsa00770 | Pantothenate and CoA biosynthesis |  |  |  |  | X |  | X | X |  |  |  |  | 3 |
| hsa04010 | MAPK signaling pathway |  |  |  |  | X | X | X |  |  |  |  |  | 3 |
| hsa04012 | ErbB signaling pathway |  |  | X |  | X |  | X |  |  |  |  |  | 3 |
| hsa04210 | Apoptosis |  |  | X |  | X | X |  |  |  |  |  |  | 3 |
| hsa04360 | Axon guidance |  | X |  |  | X |  | X |  |  |  |  |  | 3 |
| hsa04530 | Tight junction |  |  | X |  | X |  |  | X |  |  |  |  | 3 |
| hsa04664 | Fc epsilon RI signaling pathway |  |  | X |  | X | X |  |  |  |  |  |  | 3 |
| hsa04920 | Adipocytokine signaling pathway |  | X |  |  | X | X |  |  |  |  |  |  | 3 |
| hsa05222 | Small cell lung cancer |  |  | X |  | X | X |  |  |  |  |  |  | 3 |
| hsa00040 | Pentose and glucuronate interconversions |  |  |  |  | X | X |  |  |  |  |  |  | 2 |
| hsa00140 | C21-Steroid hormone metabolism |  |  |  |  | X |  | X |  |  |  |  |  | 2 |
| hsa00232 | Caffeine metabolism |  |  |  |  | X | X |  |  |  |  |  |  | 2 |
| hsa00272 | Cysteine metabolism |  |  |  |  | X |  |  | X |  |  |  |  | 2 |
| hsa00400 | Phenylalanine, tyrosine and tryptophan biosynthesis |  |  |  |  | X |  |  | X |  |  |  |  | 2 |
| hsa00520 | Nucleotide sugars metabolism |  |  | X |  | X |  |  |  |  |  |  |  | 2 |
| hsa00531 | Glycosaminoglycan degradation |  |  |  |  | X |  |  | X |  |  |  |  | 2 |
| hsa00561 | Glycerolipid metabolism |  |  |  |  | X | X |  |  |  |  |  |  | 2 |
| hsa00900 | Terpenoid biosynthesis |  |  |  |  | X |  | X |  |  |  |  |  | 2 |
| hsa04310 | Wnt signaling pathway |  |  | X |  | X |  |  |  |  |  |  |  | 2 |
| hsa04330 | Notch signaling pathway |  |  |  |  | X |  | X |  |  |  |  |  | 2 |
| hsa04370 | VEGF signaling pathway |  |  | X |  | X |  |  |  |  |  |  |  | 2 |
| hsa04910 | Insulin signaling pathway |  |  |  |  | X | X |  |  |  |  |  |  | 2 |
| hsa05211 | Renal cell carcinoma |  |  |  |  | X | X |  |  |  |  |  |  | 2 |
| hsa05214 | Glioma |  |  |  |  | X |  | X |  |  |  |  |  | 2 |
| hsa05219 | Bladder cancer |  |  |  |  | X |  |  | X |  |  |  |  | 2 |
| hsa05221 | Acute myeloid leukemia |  |  |  |  | X |  | X |  |  |  |  |  | 2 |
| hsa00051 | Fructose and mannose metabolism |  |  |  |  |  |  |  | X |  |  |  |  | 1 |
| hsa00061 | Fatty acid biosynthesis |  |  |  |  | X |  |  |  |  |  |  |  | 1 |
| hsa00130 | Ubiquinone and menaquinone biosynthesis |  |  |  |  | X |  |  |  |  |  |  |  | 1 |
| hsa00290 | Valine, leucine and isoleucine biosynthesis |  |  |  |  | X |  |  |  |  |  |  |  | 1 |
| hsa00430 | Taurine and hypotaurine metabolism |  |  |  |  | X |  |  |  |  |  |  |  | 1 |
| hsa00471 | D-Glutamine and D-glutamate metabolism |  |  |  |  | X |  |  |  |  |  |  |  | 1 |
| hsa00532 | Chondroitin sulfate biosynthesis |  |  |  |  | X |  |  |  |  |  |  |  | 1 |
| hsa00563 | Glycosylphosphatidylinositol(GPI)-anchor biosynthesis |  |  |  |  | X |  |  |  |  |  |  |  | 1 |
| hsa00565 | Ether lipid metabolism |  |  |  |  | X |  |  |  |  |  |  |  | 1 |
| hsa00601 | Glycosphingolipid biosynthesis - lacto and neolacto series |  |  |  |  | X |  |  |  |  |  |  |  | 1 |
| hsa00603 | Glycosphingolipid biosynthesis - globo series |  |  |  |  | X |  |  |  |  |  |  |  | 1 |
| hsa00643 | Styrene degradation |  |  |  |  |  |  | X |  |  |  |  |  | 1 |
| hsa00720 | Reductive carboxylate cycle (CO2 fixation) |  |  |  |  | X |  |  |  |  |  |  |  | 1 |
| hsa00785 | Lipoic acid metabolism |  |  |  |  | X |  |  |  |  |  |  |  | 1 |
| hsa00920 | Sulfur metabolism |  |  |  |  |  | X |  |  |  |  |  |  | 1 |
| hsa00950 | Alkaloid biosynthesis I |  |  |  |  | X |  |  |  |  |  |  |  | 1 |
| hsa01040 | Biosynthesis of unsaturated fatty acids |  |  |  |  |  |  |  |  |  |  | X |  | 1 |
| hsa03060 | Protein export | X |  |  |  |  |  |  |  |  |  |  |  | 1 |
| hsa04020 | Calcium signaling pathway |  |  |  |  | X |  |  |  |  |  |  |  | 1 |
| hsa04115 | p53 signaling pathway |  |  |  |  | X |  |  |  |  |  |  |  | 1 |
| hsa04130 | SNARE interactions in vesicular transport |  |  |  |  | X |  |  |  |  |  |  |  | 1 |
| hsa04140 | Regulation of autophagy |  |  |  |  | X |  |  |  |  |  |  |  | 1 |
| hsa04540 | Gap junction |  |  |  |  | X |  |  |  |  |  |  |  | 1 |
| hsa04612 | Antigen processing and presentation |  |  |  |  | X |  |  |  |  |  |  |  | 1 |
| hsa04620 | Toll-like receptor signaling pathway |  |  |  |  | X |  |  |  |  |  |  |  | 1 |
| hsa04670 | Leukocyte transendothelial migration |  |  |  |  | X |  |  |  |  |  |  |  | 1 |
| hsa04730 | Long-term depression |  |  |  |  | X |  |  |  |  |  |  |  | 1 |
| hsa04740 | Olfactory transduction |  |  |  |  | X |  |  |  |  |  |  |  | 1 |
| hsa04742 | Taste transduction |  |  |  |  | X |  |  |  |  |  |  |  | 1 |
| hsa04912 | GnRH signaling pathway |  |  |  |  | X |  |  |  |  |  |  |  | 1 |
| hsa04916 | Melanogenesis |  |  |  |  | X |  |  |  |  |  |  |  | 1 |
| hsa04950 | Maturity onset diabetes of the young |  |  |  |  | X |  |  |  |  |  |  |  | 1 |
| hsa05010 | Alzheimer's disease |  |  |  |  | X |  |  |  |  |  |  |  | 1 |
| hsa05012 | Parkinson's disease |  |  |  |  | X |  |  |  |  |  |  |  | 1 |
| hsa05014 | Amyotrophic lateral sclerosis (ALS) |  |  |  |  | X |  |  |  |  |  |  |  | 1 |
| hsa05110 | Vibrio cholerae infection |  |  |  |  | X |  |  |  |  |  |  |  | 1 |
| hsa05120 | Epithelial cell signaling in Helicobacter pylori infection |  |  |  |  | X |  |  |  |  |  |  |  | 1 |
| hsa05130 | Pathogenic Escherichia coli infection - EHEC |  |  |  |  | X |  |  |  |  |  |  |  | 1 |
| hsa05131 | Pathogenic Escherichia coli infection - EPEC |  |  |  |  | X |  |  |  |  |  |  |  | 1 |
| hsa05210 | Colorectal cancer |  |  |  |  | X |  |  |  |  |  |  |  | 1 |
| hsa05212 | Pancreatic cancer |  |  |  |  | X |  |  |  |  |  |  |  | 1 |
| hsa05213 | Endometrial cancer |  |  |  |  | X |  |  |  |  |  |  |  | 1 |
| hsa05215 | Prostate cancer |  |  |  |  | X |  |  |  |  |  |  |  | 1 |
| hsa05216 | Thyroid cancer |  |  |  |  | X |  |  |  |  |  |  |  | 1 |
| hsa05217 | Basal cell carcinoma |  |  |  |  | X |  |  |  |  |  |  |  | 1 |
| hsa05218 | Melanoma |  |  |  |  | X |  |  |  |  |  |  |  | 1 |
| hsa05220 | Chronic myeloid leukemia |  |  |  |  | X |  |  |  |  |  |  |  | 1 |
| hsa05223 | Non-small cell lung cancer |  |  |  |  | X |  |  |  |  |  |  |  | 1 |
| TOTAL | | 1 | 18 | 41 | 3 | 122 | 50 | 58 | 54 | 0 | 25 | 1 | 0 |  |
