# Supplementary material for: Bayesian Pathway Analysis of Cancer Microarray Data
Source: PLoS One. 2014 Jul 18;9(7):e102803. doi: 10.1371/journal.pone.0102803 (PMC4103872; doi:10.1371/journal.pone.0102803)
Supplement: Table S5 — Overlap of active pathways identified by the new and old BPA systemson real cancer microarray data sets. (DOCX) [file pone.0102803.s005.docx]

**Table S5.** Overlap of active pathways identified by the new and old BPA systemson real cancer microarray data sets.

| **GEO #** | **Cancer Type** | **Affymetrix Chip Type** | **# of Samples** | **# of Significant Pathways** | | |
| --- | --- | --- | --- | --- | --- | --- |
|  |  |  |  | **New BPA** | **old BPA** | **overlap** |
| GSE 7476 | bladder | HG-U133 Plus 2 | 12 (9C, 3N) | 57 | 1 | 0 |
| GSE 12907 | brain | HG-U133A | 25 (21C, 4N) | 81 | 18 | 7 |
| GSE 15824 | brain | HG-U133 Plus 2 | 35 (30C, 5N) | 46 | 41 | 13 |
| GSE 8977 | breast | HG-U133 Plus 2 | 22 (7C, 15N) | 16 | 3 | 0 |
| GSE 22544 | breast | HG-U133 Plus 2 | 18 (14C, 4N) | 66 | 122 | 58 |
| GSE 41328 | colon | HG-U133 Plus 2 | 20 (10C, 10N) | 36 | 50 | 13 |
| GSE 14520 | liver | HG-U133A 2 | 43 (22C, 21N) | 59 | 54 | 28 |
| GSE 14323 | liver | HG-U133A 2 | 66 (47C, 19N) | 77 | 58 | 23 |
| GSE 10799 | lung | HG-U133 Plus 2 | 19 (16C, 3N) | 58 | 0 | 0 |
| GSE 14407 | ovarian | HG-U133 Plus 2 | 24 (12C, 12N) | 5 | 25 | 0 |
| GSE 3678 | thyroid | HG-U133 Plus 2 | 14 (7C, 7N) | 4 | 1 | 1 |
| GSE 6004 | thyroid | HG-U133 Plus 2 | 18 (14C, 4N) | 10 | 0 | 0 |
